# Apparent Diffusion Coefficient analysis of encephalitis: A comparative study with topographic evaluation and conventional MRI findings 

**DOI:** 10.12669/pjms.323.10030

**Published:** 2016

**Authors:** Ahmet Katirag, Mehtap Beker-Acay, Ebru Unlu, Hayri Demirbas, Nese Demirturk

**Affiliations:** 1 Ahmet Katirag, MD. Resident Assistant, Department of Radiology, Afyon Kocatepe University Faculty of Medicine, Afyonkarahisar, Turkey; 2 Mehtap Beker-Acay, MD. Assistant Professor, Department of Radiology, Afyon Kocatepe University Faculty of Medicine, Afyonkarahisar, Turkey; 3 Ebru Unlu, MD. Assistant Professor, Department of Radiology, Afyon Kocatepe University Faculty of Medicine, Afyonkarahisar, Turkey; 4 Hayri Demirbas M.D. Assistant Professor, Department of Neurology, Afyon Kocatepe University Faculty of Medicine, Afyonkarahisar, Turkey; 5 Nese Demirturk MD. Associate Professor, Department of Infectious Diseases, Afyon Kocatepe University Faculty of Medicine, Afyonkarahisar, Turkey

**Keywords:** Encephalitis, Difffusion weighted imaging, Magnetic resonance imaging

## Abstract

**Objective:**

Our purpose was to reveal the efficiency of diffusion weighted imaging (DWI) in the diagnosis of encephalitis, and to determine the relation between the apparent diffusion coefficient (ADC) values, the onset of the clinical symptoms, and the lesion extent.

**Methods:**

Conventional magnetic resonance imaging (MRI) was performed in 17 patients with encephalitis diagnosed on the basis of laboratory, clinical and radiologic findings during 2009 and 2015. Based on the duration between the onset of the symptoms and the brain MRI findings, the patients were divided into three groups. ADC values of the encephalitis lesion, the lesions’ topographic analysis score, deep gray matter involvement, patients’ clinical situation and the duration of the arrival to the clinic was examined.

**Results:**

Mean ADC values were 0,988±0,335 x10^-3^ mm^2^/s in group I (0-2 days), 1,045±0,347 x10^-3^ mm^2^/s in Group-II (3-7 days), 1,451±0,225 x10^-3^ mm^2^/s in Group-III (8 days and over). The relation between the ADC values and the duration of the arrival, topographic analysis score, the relation between the patients’ clinical situation and the deep gray matter involvement were found to be statistically significant. The deep gray matter involvement was demonstrated more clearly by FLAIR images when compared with DWI.

**Conclusion:**

Conventional MRI sequences may be insufficient in showing the encephalitis lesion. DWI must be added to the imaging modalities immediately in the cases suspected of having encephalitis.

## INTRODUCTION

Encephalitis is a life-threatening inflammatory disease of the brain which may be seen at any age. Various agents, as viral agents being in the first line, cause this disease.^1^-^3^ Herpes Simplex Virus (HSV) is the most common cause of sporadic acute encephalitis worldwide.^4^ Early diagnosis and early administration of acyclovir are crucial for decreasing mortality and morbidity rates.^5^ Role of imaging methods in the management of encephalitis remains limited to confirmation of diagnosis and follow-up of complications.^6^

Magnetic resonance imaging (MRI) is widely accepted as a sensitive imaging method used in detection of early changes in encephalitis. Routine MRI sequences include T1, T2 and Fluid Attenuated Inversion Recovery (FLAIR) images which are applied in order to define abnormal areas related with viral encephalitis. These sequences, however, may remain insufficient in demonstrating encephalitic lesions at very early stages.^7^,^8^

Diffusion-weighted imaging (DWI) is ever-increasingly used in various diseases, mostly in early diagnosis of cerebral ischemia; however its role in other conditions like infections is in phase of discovery.^9^-^11^ Increasing sensitivity of DWI concerning viral encephalitis has been demonstrated in some studies which were performed with limited number of patients.^12^,^13^ In this study, we aimed to study the effectiveness of brain DWI in the detection of encephalitic lesions at early stages.

## METHODS

Patients which came to department of radiology in our hospital with the pre-diagnosis of encephalitis were enrolled in the study. MRI work-up with standard sequences were performed to these patients between November 2009 and April 2015. Approval from ethics committee of the university was received for the study (2015/09-234).

Diagnosis of encephalitis was reached through evaluation of laboratory analyses of blood and cerebrospinal fluid (CSF), culture results, clinical and radiological findings of the patients by the neurology and infectious diseases departments of our hospital. Cases in which ADC and FLAIR sequence images were missing and images were obtained through inappropriate methods were excluded from the study.

**Fig.1:**
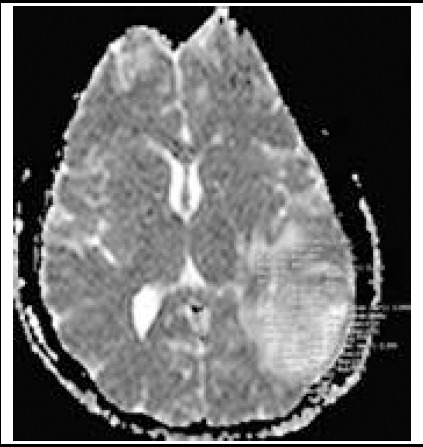
Measurement of mean signal intensity of encephalitis area by freehand ROIs on b1000 images of ADC map.

MRI images of the patients were evaluated retrospectively. ADC images which are present in routine cranial MRI protocol and are formed automatically by the MRI unit were used. Measurement was performed through homogenous placing of region of interest (ROI) onto areas with signal alteration as 2-3 mm^2^ of ROI corresponding to 1 cm^2^ of area, regardless of white and gray matters (Fig.1). Approximately 3-50 ROI areas were used for each patient as proportionate to brain parenchymal involvement. ADC values were recorded by dividing each signal intensity by 1000 in order to obtain values as unit ADCx10^-3^ mm^2^/s. Arithmetic mean was calculated through summation of each ROI value.

Patients were grouped in the consideration of the time from being symptomatic to cranial MRI work-up. Those patients for which this duration was between 0-2 days were classified as Group- I, ones for which this duration was between 3-7 days were classified as Group-II and patients for which this duration was 8 or more were classified as Group-III.^14^ In order to evaluate the effectiveness of parenchymal involvement, topographical analysis scores which were defined in the study conducted by Renard et al. were used.^15^ According to this, in consideration of axial slices of each hemisphere a total of 9 points for temporal lobe, 9 points for frontal lobe, three points for parietal lobe, two points for occipital lobe and two points for deep gray matter structures were attributed.

For areas with hemorrhage, ROI was not placed and this area was carefully excluded from the area of interest. Involvement of deep brain structures were recorded separately. Follow-up MRI images of the patients were examined and ADC values were recorded. Follow-up MRI images of four patients could not be found in the archive. One patient in group I did not have FLAIR sequence and he was excluded from evaluation. Evaluation of clinical presentations of patients was based upon status of consciousness. Clinical presentations of patients without deterioration of consciousness were classified as “fine” and clinical presentations of patients with deterioration of consciousness were classified as “moderate-poor”.

The relationship among mean ADC values and topographical analysis scores at the admission of the patients with admission time, extensiveness of parenchymal involvement, deep gray matter involvement and clinical findings was examined.

Statistical analyses of obtained data were performed using SPSS 18 program. In analysis of obtained data, along with descriptive statistics, relationship between initial ADC values and the involvement of brain parenchyma were analysed using Spearman’s Rho correlation test. Relationship among mean ADC values and the groups was analysed using Kruskal Wallis test. Since the relationship among groups was found to be statistically significant, relationship among groups was examined with post hoc (Conover-Iman test of multiple comparison) test. Relationship between admission time and involvement of brain parenchyma was examined with Man Whitney U test. The relationship between topographical analysis score and clinical presentation was examined with Kruskal Wallis test. P<0.05 was considered as significant.

## RESULTS

A total of 17 patients whose ages ranged from 0 to 85 years old with a mean age of 37 were evaluated. According to the gender of the patients 15 (88.2%) were male and 2 (11.8%) were female. When patients were classified for admission time; there were 5 patients in Group-I (0-2 days), 6 patients in each of Group-II (3-7 days) and Group-III (8 days and more) (Table-I).

**Table-I:** Patients’ time onset from the beginning of the symptoms, first apparent diffusion coefficient (ADC) values and topographic analysis scores.

*Patients (n)*	*Admission time (days) (group)*	*ADC value on first MRI (x10^-3^ mm^2^/s)*	*Topographic analysis score*
1	8 (3)	1.435	6
2	11 (3)	1.597	2
3	7 (2)	1.236	13
4	2 (1)	1.279	5
5	4 (2)	1.040	21
6	1 (1)	1.069	8
7	3 (2)	1.529	1
8	22 (3)	1.711	1
9	4 (2)	0.477	15
10	1 (1)	1.094	14
11	15 (3)	1.111	15
12	9 (3)	1.272	3
13	5 (2)	0.944	16
14	56 (3)	1.580	11
15	1 (1)	0.408	7
16	2 (1)	1.093	10
17	3 (2)	1.046	6

**Fig.2:**
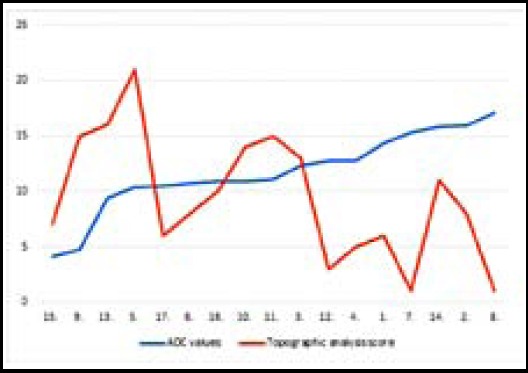
Topographic analysis score and ADC values of the patients. Vertical line represents ADC values (x10^-3^ mm^2^/s) and the horizontal line represents the day of admission.

Relationship among mean ADC values and groups was found to be significant (p<0.05). When the relationship among groups was considered; whereas no statistically significant difference was found between group I and II, a statistically significant difference was found between group 2 and 3 (p=0.042). Although there was an obvious difference in ADC values between Group-1 and 3, it was not found to be statistically significant due to low number of patients (p=0.085) (Table-II). Relationship between admission time and brain parenchymal involvement was not statistically significant (p:0.421) (Table-II). When the relationship between initial ADC values of the patients and the parenchymal involvement was examined, a significant moderate inverse correlation was found between them (p<0.05, r= - 0.545) (Fig.2).

**Table-II:** The relationship between first apparent diffusion coefficient (ADC) values (x10^-3^ mm^2^/s) and topographic analysis scores among patient groups.

	*Group-1*	*Group 2*	*Group 3*	*P value*
Mean ADC value±SD	0.988±0.335	1.045±0.347	1.451±0.225	0.025
Mean topographic analysis score±SD	9.83±3.42	11.4±7.26	7.3±5.16	0.421

When the relationship between topographical analysis scores and clinical presentation of the patients was examined; topographical analysis score was found to be 9.37±6.69 in 8 patients with fine clinical presentation and to be 9.44±4.97 in 9 patients with moderate-poor clinical presentation. However no statistically significant difference was found (p>0.05). Relationship among clinical presentation of the patients and deep gray matter involvement was found to be statistically significant (p:0.009). All the patients with moderate-poor clinical presentation, but only three out of 11 patients with fine clinical presentation had deep gray matter involvement.

**Fig.3:**
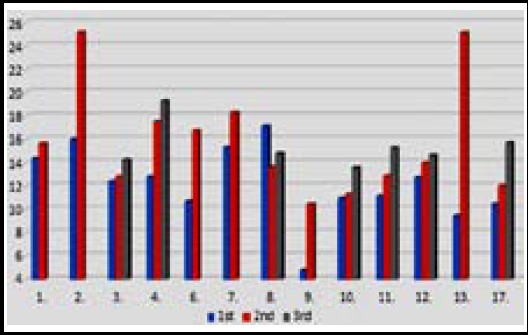
Initial & follow-up ADC values of the patients (x10^-3^ mm^2^/s) Horizontal line demonstrates number of the patients.

Thirteen of 17 patients had follow-up MRI images from different time intervals. It was observed that in ADC measurements performed on follow-up MRI images were eventually increased (Fig.3).

When topographical analysis scoring was performed on DWI and FLAIR images, no significant difference was observed in terms of lesional extensiveness, however 1 score difference was found in 4 patients due to the superiority of FLAIR sequences in thalamic involvement. However, lesions seemed to be different in DWI and FLAIR sequences according to the time in terms of resolution. In group I; whereas signal changes seemed more obvious in DWI, in one of the patients FLAIR images were superior to DWI, in 2 patients DWI and FLAIR have similar resolution. In Group-II; in 2 patients DWI and in the remaining 4 patients FLAIR had higher resolution. In Group-III; lesion seemed to be obvious in FLAIR images in all of 6 patients. Statistical analysis could not be performed due to limited number of the patients.

## DISCUSSION

Central nervous system infection represents itself with different symptoms which usually present as meningitis or encephalitis. Fate of the disease may range from a benign clinical presentation to encephalitis in which life-threatening neurological symptoms develop due to the factors specific to the host and organism.^16^

Role of imaging methods in the management of encephalitis remains limited to confirmation of diagnosis and follow-up of complications. Imaging patterns demonstrate lesion extension, type of edema and frequently contribute to determine the underlying type of infection; however, clinical and laboratory analyses are required for characterization of infectious agents.^6^

**Fig.4:**
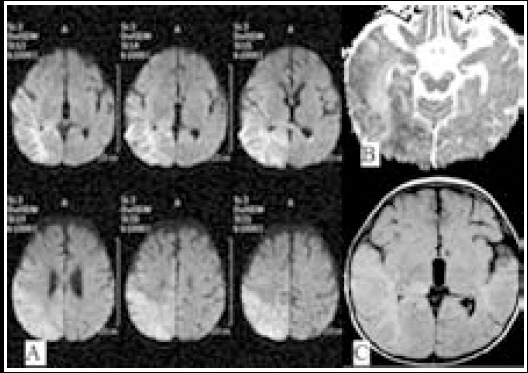
3 year-old male patient, on the 5th day of admission: On DWI (image A), ADC series (image B) and FLAIR image (image C) restricted diffusion on the right hemisphere involving cortico-subcortical and deep white mater areas. Hyperintense signal is observed in FLAIR serial but conspicuity of signal alterations is best depicted on DWI due to the early examination (secondary to predominance of cytotoxic edema).

**Fig.5:**
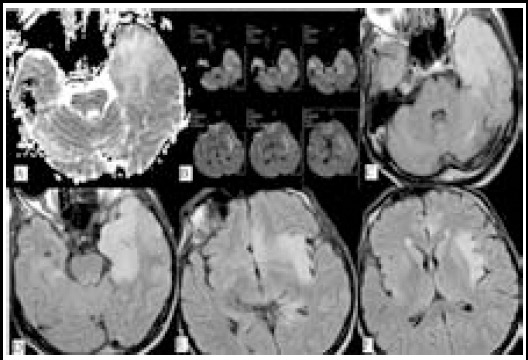
53 year-old male patient with encephalitis, on the 15^th^ day of admission: On ADC sequence (image A), DWI (image B) and axial FLAIR sequences (images C-F), hyperintensity extending from left temporal lobe up to insula and cingulate gyrus is being observed but conspicuity of signal alterations is best depicted on FLAIR images.

MRI is obviously superior to other radiological modalities in demonstrating brain parenchyma.^17^ Signal changes in DWI may be related with pathological changes occurring after viral invasion. In acute stage, there are congestion, perivascular cell infiltration named as “perivascular cuffing” and thrombus formation pathologically.^17^ Possible mechanism regarding increased signal in DWI is the presence of cytotoxic edema in primarily affected gray matter neurons^18^,^19^ (Fig.4). In late acute and early subacute stages, vasculitis and perivascular cell infiltration decrease, and thus the severity of diffusion restriction diminishes and ADC values begins to increase^13^ (Fig.5). This stage is also a process which is accompanied by vasogenic edema which becomes visible in T2 imaging. In chronic stage, necrosis and demyelination begin and these are responsible for increased signal on T2 sequence, as well as higher ADC values.^12^

In the study conducted by Kiroglu et al. in which they quantitatively evaluated DWI and conventional MRI in determination of the encephalitic lesions in 18 patients, they found that DWI was superior to conventional series in early-stage encephalitic lesions and conventional MRI was superior to DWI in late-stage disease.^12^ In our study, as similar to the literature, DWI was found to be superior due to cytotoxic edema in early stage in most of the patients, and FLAIR images were superior in late-stage because it was replaced by vasogenic edema.

Prakash et al. reported that T2 imaging was superior to DWI in two patients with encephalitis whose images were taken at 3-7 weeks after neurological symptoms were obvious.^13^ In another study, it was defined that FLAIR and T2 imaging have similar signal characteristics and FLAIR images are even superior.^6^ In this study T2 images were not evaluated, but we demonstrated that FLAIR imaging is superior to DWI in late-stage, similar to the aforementioned study.

Tsuchiya et al. reported that some patients with encephalitis presented with extensive vasogenic edema in acute stage and FLAIR imaging was equal or superior to DWI in these patients.^2^ In this study, in similar, there was marked vasogenic edema in acute stage and in 5 patients out of 11, this was observed in FLAIR images. In the study by Sener RN et al., there were 2 definite patterns in DWI as being vasogenic and cytotoxic edema and the effect of these patterns of edema on prognosis was investigated. Whereas clinical presentations of patients having ADC values accordant with cytotoxic edema were poor, two patients with high ADC values exhibited a rapid recovery following the treatment. It was suggested that cytotoxic edema in Herpes Simplex encephalitis (HSE) was obviously related with the prognosis of the patient.^20^ In our study, there were two patients having very low ADC values and clinical presentations of these patients were moderate-poor. However, ADC values of other patients with moderate-poor clinical status were not low as to be accordant with those in that study. In addition, an inverse relationship was found among ADC values and extensiveness of involved areas. According to this, ADC values seem to be not only related with poor clinical presentation, but also with more extensive involvement of parenchyma. Additionally, it was concluded in our study that involvement of brain deep gray matter made the clinical status significantly worse.

In the study of Renard et al. in which they compared DWI and FLAIR imaging in initial and follow-up MRIs of 11 patients with HSE, topographical analysis scoring was applied, as in our study.^15^ DWI scores of the patients in acute stage were found to be higher than FLAIR imaging, and in late stage FLAIR imaging scores were found to be higher than DWI. “Cut-off” score values for single hemisphere for bilateral involvement for FLAIR imaging and DWI were found to be 8/50 and 9/50, respectively. On the contrary, FLAIR imaging was found to be superior to DWI in demonstrating thalamic involvement, including the early stage.^15^ No significant score difference was observed between FLAIR imaging and DWI in terms of lesion extensiveness and FLAIR imaging seemed to be obviously superior to DWI in late stage in terms of lesion resolution. In our study, in 6 of 17 patients with brain deep gray matter involvement, FLAIR imaging was obviously superior to DWI in depicting this, regardless of group classification in concordance with this study. In our study, one of the patients had bilateral involvement. For this patient, single hemisphere score was recorded as two and one for the other hemisphere and it does not correspond to the bilaterality “cut-off” score defined in that study.

### Limitations of our study

We had limited number of patients. Due to the retrospective design of the study, encephalitis diagnosis was based on clinical, laboratory, CSF and radiological findings. Advanced microbiological examinations such as polymerase chain reaction (PCR) analysis may not be available in our country sometimes. Nevertheless, we are in thought of that our results will be a guide in approach to this destructive pathology that may be encountered in daily life at any time.

## CONCLUSION

Radiological imaging has to be considered for exclusion of an intracranial pathology in a patient that admits with presentation of acute encephalitis. Early diagnosis and rapid treatment are essential in this disease in order to reduce mortality and morbidity. Therefore, DWI must be added to the imaging modalities in the first touch of the cases suspected of having encephalitis.
